# A novel live-cell imaging system reveals a reversible hydrostatic pressure impact on cell-cycle progression

**DOI:** 10.1242/jcs.212167

**Published:** 2018-08-06

**Authors:** Holly R. Brooker, Irene A. Gyamfi, Agnieszka Wieckowska, Nicholas J. Brooks, Daniel P. Mulvihill, Michael A. Geeves

**Affiliations:** 1 School of Biosciences, University of Kent, Canterbury, Kent CT2 7NJ, UK; 2 Department of Chemistry, Imperial College London, London SW7 2AZ, UK

**Keywords:** Fission yeast, Live-cell imaging, Microscopy, Cell synchronisation

## Abstract

Life is dependent upon the ability of a cell to rapidly respond to changes in the environment. Small perturbations in local environments change the ability of molecules to interact and, hence, communicate. Hydrostatic pressure provides a rapid non-invasive, fully reversible method for modulating affinities between molecules both *in vivo* and *in vitro*. We have developed a simple fluorescence imaging chamber that allows intracellular protein dynamics and molecular events to be followed at pressures <200 bar in living cells. By using yeast, we investigated the impact of hydrostatic pressure upon cell growth and cell-cycle progression. While 100 bar has no effect upon viability, it induces a delay in chromosome segregation, resulting in the accumulation of long undivided cells that are also bent, consistent with disruption of the cytoskeletons. This delay is independent of stress signalling and induces synchronisation of cell-cycle progression. Equivalent effects were observed in *Candida albicans*, with pressure inducing a reversible cell-cycle delay and hyphal growth. We present a simple novel non-invasive fluorescence microscopy-based approach to transiently impact molecular dynamics in order to visualise, dissect and study signalling pathways and cellular processes in living cells.

## Abstract (precis)

**Summary:** Development of a simple fluorescence imaging chamber allowing observation of intracellular protein dynamics and molecular events in living cells at pressure up to 200 bar.

## INTRODUCTION

All life is dependent upon the ability of a cell to rapidly respond to changes in its environment through modulation of diverse signalling pathways. Small perturbations in local environments change the ability of molecules to interact and, hence, communicate. Hydrostatic pressure provides a rapid non-invasive and fully reversible method to modulate the affinities between molecules both *in vivo* and *in vitro*.

Hydrostatic pressure is a powerful tool to perturb protein–protein and protein–ligand interactions in complex environments. It has been widely used to study proteins and membranes in solution (see, e.g. Barriga et al., 2016; Brooks et al., 2011; Coates et al., 1985; Eccleston et al., 1988) but less so in cellular systems. Yet, this benign approach is well-tolerated by cells. Little compression (∼1%) takes place as water is inherently incompressible at the pressure used here, i.e. 200 bar (which equals 20 MPa) (Kell, 1975). Instead changes in hydrostatic pressure induce their effect on proteins through changes in the water structure (hydration shells) (Kitching, 1972). As such, it is an ideal technique to perturb systems that are close to a 1:1 thermodynamic balance – and this applies to many sensory and signalling pathways. Pressure can be applied to living cells and released within <1 sec, and is transmitted through complex structures at the speed of sound. Rapid readjustment to the new pressure, therefore, depends upon the response of the cell. It, thus, has significant advantages over other methods that can alter cellular dynamics, such as drugs or changes in temperature, both of which can induce slow response and a slow recovery in addition to the induction of stress checkpoints.

Although effects of pressure on the cell cycle have been reported before, only very high pressure (≥700 bar) had usually been applied for only brief periods before releasing it to 1 bar in order to observe cell behaviour or response (George et al., 2007). Exposing cells to extreme high pressures even for a short period can have a dramatic impact on cell viability (George et al., 2007; Arai et al., 2008) and provides the basis for industrial sterilisation protocols (Balasubramaniam et al., 2015; Follonier et al., 2012). Earlier high-resolution studies have demonstrated that increased hydrostatic pressure affects membrane permeability (Otter and Salmon, 1979; Roberts et al., 1998) and the structural organisation of cytoskeleton (Begg et al., 1983; Marsland, 1965; Salmon, 1975a,b; Salmon et al., 1976; Tilney et al., 1966). In these studies, live-cell imaging was restricted to reports regarding changes in cell morphology and organelles by using transmitted light microscopy methods. Precise protein localisation relied on fixing samples at high pressure or immediately after pressure release. To date, dynamics of individual proteins have not been followed in live cells while held at significant pressure. This is largely because of the difficulty in designing windows that allow high-resolution fluorescence imaging, yet are able to withstand the pressure involved. We have now constructed a pressure cell that can image fluorescently labelled molecules in living cells at 200 bar without detectable optical distortion. The system has a resolution of ∼400 nm and allows the dynamics of individual proteins to be followed in living cells held at pressure.

We demonstrate here that much more can be gleaned about how pressure perturbs cell signalling, when live cells with readily available fluorescent markers are imaged during moderate increases in pressure (1–100 bar) that do not impact viability. These pressures are ideal to perturb signalling pathways because they only affect reactions that occur together with very large changes in volume, e.g. actin or tubulin polymerisation (Davis and Gutfreund, 1976; Kitching, 1972; Swezey and Somero, 1985) or in systems showing moderate changes in volume when poised near a 1:1 equilibrium or steady-state position (Geeves and Pearson, 2013). These latter reactions include Ca^2+^- and nucleotide-binding reactions, as well as conformational changes of proteins (Geeves and Gutfreund, 1982; Pearson et al., 2008). High pressure (>200 bar) is lethal to most prokaryotic and eukaryotic cells.

We used the genetically tractable fission yeast model system and this simple pressure chamber to study the impact pressure has upon cellular functions. The simple rod-like shape and size of fission yeast allows live-cell imaging studies of diverse cellular processes. Upon pressure application of 100 bar, mid-log fission yeast cells became elongated and underwent a cell-cycle delay. While actin patch dynamics and endocytosis are unaffected, fluorescent protein labelling revealed a significant delay in chromosome segregation and subsequent cytokinesis. Intriguingly, the growth of the yeast culture became synchronised with respect to cell-cycle progression at 100 bar. We were able to reversibly arrest cell division and induce synchronisation of cell-cycle progression. The pressure failed to induce a mitogen-activated stress response within the yeast cells. For example, while the stress activation pathway kinase, p38, was seen to import into the nucleus in response to a 10°C change in temperature, this signalling protein remained cytoplasmic upon exposure to 100 bar hydrostatic pressure. We also examined the impact pressure has upon the cell-cycle progression of the pathological yeast *Candida albicans*. Like fission yeast cells, *C. albicans* underwent cell-cycle arrest when pressure was applied at 100 bar, and hyphal growth was also induced. Normal vegetative growth was rapidly restored upon returning to atmospheric pressure. Thus, we describe here a novel mechanism to rapidly and reversibly disrupt molecular interactions without impacting on cell viability, and provide an exciting opportunity to dissect cell growth and signalling pathways in living cells.

## RESULTS

The effects of pressure on growth of bacteria, yeast cells and animals has been well documented (Demazeau and Rivalain, 2011; Larson et al., 1918), and pressures of above 200 bar result in cell death. Here, we were interested in the effects of moderate elevated pressure that perturbs cell growth and signalling but does not result in cell death. Initial control studies used a static pressure chamber that could maintain high pressure for several hours but the cells could not be observed directly while held at high pressure. Fission yeast cells, in mid-log phase at 25°C, were placed in the pressure chamber and exposed to elevated pressure for times between 1 and 24 h before pressure was returned to 1 bar, and samples were collected for viewing using standard microscopy or were plated out to assess viability.

**Fig. 1.**
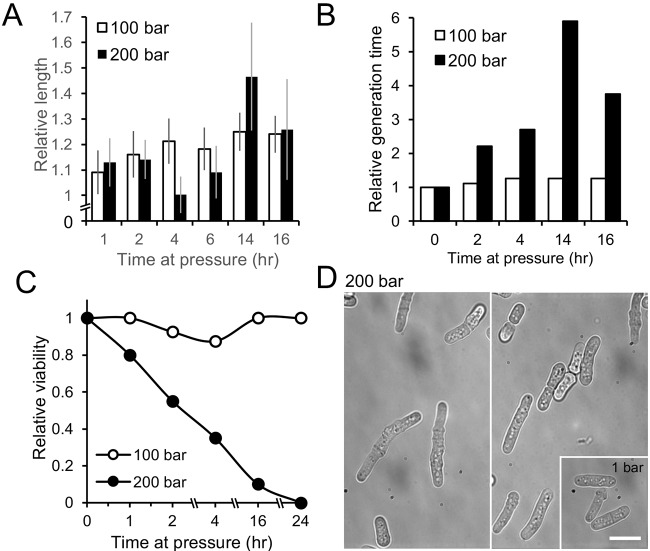
**Impact of high pressure on fission yeast.** (A-C) Fission yeast cells were cultured at 25°C under pressures of 1, 100 or 200 bar for different times. Calculated were the cell length (A), generation time (B) and cell viability (C) relative to control cells that were kept at 1 bar. Data represent averages of >100 cells for each condition and time point. Each experiment was repeated three times. Error bars represent ±s.e.m. Student’s *t*-test were applied to indicate significant differences (99% level of confidence) in cell length (A), generation time (B) and viability of cells when incubated at either 100 or 200 bar pressure for >2 h. (D) Different fields of view of cells treated the same way. Micrographs illustrating bent and long cell physiology of cells immediately fixed after they had been incubated at 200 bar for 14 h. Inset show equivalent for cells cultured for same period at 1 bar pressure. Scale bar:10 µm (all three micrographs).

Exposure to 100 bar for up to 24 h had no discernible effect on cell viability once returned to 1 bar (Fig. 1C). In contrast, 24 h exposure to high pressure (200 bar) reduced cell viability to zero. Shorter exposure time reduced viability almost linearly over the first 4 h only (∼20% per hour; Fig. 1C). This was consistent with previous observations that short bursts of very high pressure (≥700 bar) have a dramatic impact upon cell viability (George et al., 2007; Arai et al., 2008). Observations of the fixed cells after exposure to pressure indicated that relative cell length increased 1.4 fold (to 15 µm) after 4 h at 100 bar (Fig. 1A) and then remained fairly constant. Exposure to 200 bar resulted in an increased variation in cell length. Exposure to 100 bar resulted in only a small (∼25%) increase in the estimated doubling time of the cells (hereafter referred to as generation time), whereas exposure to 200 bar caused a dramatic increase in generation time (Fig. 1B). Cells that had been kept at 200 bar for 14 h (peak of increased length and generation time) followed by immediate aldehyde fixation are shown in Fig. 1D. They have a bent rod shape with lengths often more than twice that of the normal cell.

**Fig. 2.**
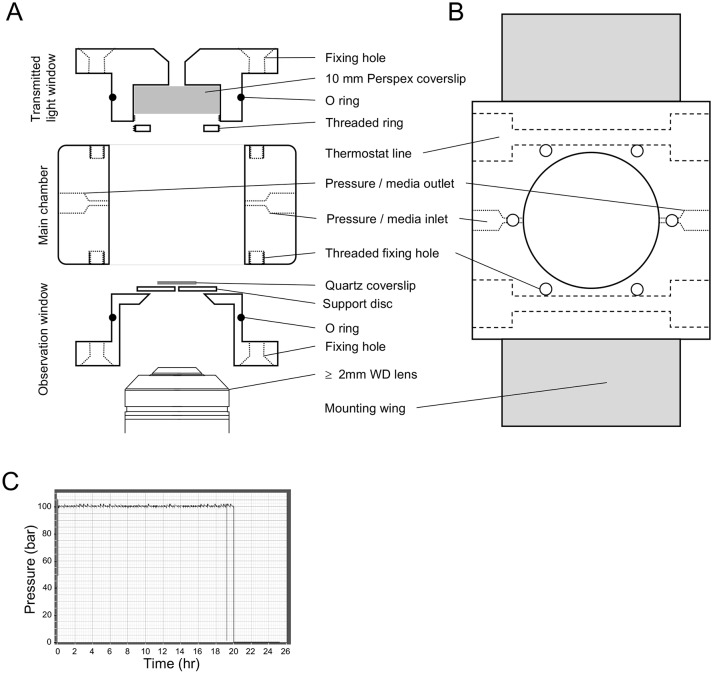
**Fluorescence microscopy pressure chamber.** (A,B) Schematic diagram showing a cross section (A) and overhead (B) view of the high-pressure imaging chamber. (C) A typical overnight pressure trace demonstrating long-term maintenance and stability of 100 bar pressure within the imaging chamber system.

The changes reported here are intriguing, but to understand what happens to the cell at pressure is difficult without direct observation of cells that grow under pressure. This is why we designed a high-pressure chamber with windows that allow direct observation of the yeast cells at elevated pressure. The key aim was to design a window able to withstand the high pressure force on the window and, at the same time, keep the working distance between lens and sample to a minimum (<2 mm) in order to allow high-resolution imaging. Our design is shown in Fig. 2 (and Fig. S1) and described in the Materials and Methods. Using this system in conjunction with a computer-controlled high-pressure pump to add medium allows to apply rapid changes in the hydrostatic pressure (i.e. increases from 1–200 bar within 2 s), followed by maintaining stable pressure for >20 h before rapid release of pressure.

**Fig. 3.**
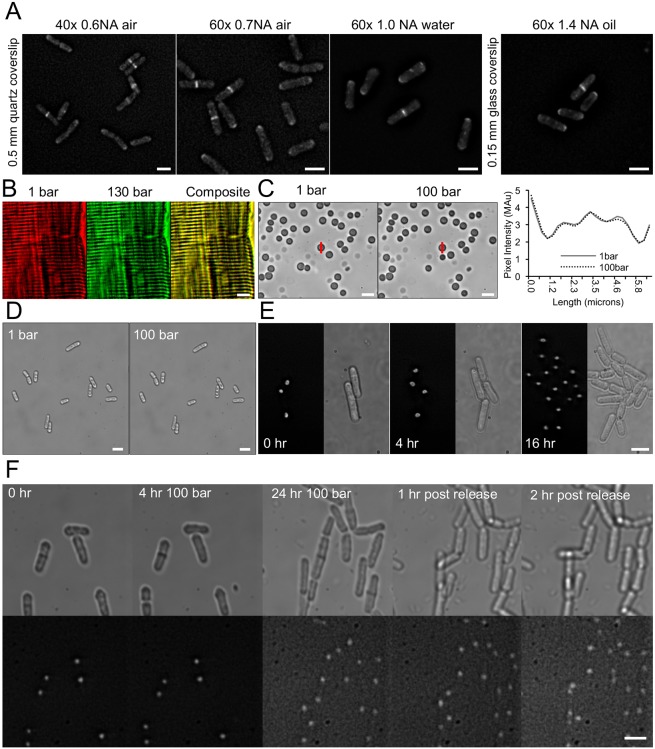
**Image quality and live-cell imaging.** (A) Micrographs of live *cam1-YFP* fission yeast cells in the pressure chamber mounted onto 0.5 mm quartz or 0.15 mm glass coverslips. Lenses with differing working distance and numerical aperture values were used as indicated. (B) Images of a rabbit muscle sarcomere mounted within the pressure chamber. Images were taken at a pressure of 1 bar (red) or 130 bar (green), using 1 mm borosilicate glass windows. The merged image (composite; yellow) shows no distortion of image across the field of view, the precise sarcomere pattern is maintained. (C) Images of porcine red blood corpuscles (left) mounted in the pressure chamber. Images were taken at pressures of 1 and 100 bar, using the same windows as in B. The line profile (red vertical line) of the same cell is shown in the graph (right), indicating that hydrostatic pressure does not compress or distort membrane structures. (D) Images of *S. pombe* cells at 1 and 100 bar pressure show unaltered cells. (E,F) Time-lapse images of *S*. *pombe hht-gfp* cells cultured in the pressure chamber showing GFP fluorescence (images on the left in E, bottom images in F) and transmitted light (images on the right in E, top images in F) under pressure of 1 bar (E) or 100 bar (F) for 0, 4 and 24 h before release to 1 bar for 2 h. Scale bars: 10 µm.

We explored several different distance lenses to image fission yeast cells (illustrated in Fig. 3A). Images were captured by using a 0.5 mm-thick quartz coverslip (window) in combination with a 40×0.6NA air lens, 60×0.7 NA air lens or a 1.0 NA water lens. A thinner 0.15 mm glass coverslip was used with a 60×1.4 NA oil lens. Using fluorescently labelled calmodulin (Cam1), an established marker of enodcytosis and polarised cell growth (Fig. 3A), fluorescence images of *cam1-YFP* fission yeast all showed the contractile ring just before cell division and an accumulation of Cam1-YFP foci at the growing tips of the cell during interphase. All images were collected at a pressure of 1 bar and demonstrate the intrinsic imaging performance of the system.

Exposure of the thin windows to high pressure was expected to distort the window shape and, indeed, the microscope required refocussing after the chamber had been pressurised; however, thereafter the image remained stable and no further refocussing was required beyond the usual. It was important to evaluate the image for distortion at elevated pressure. Fig. 3B shows images of a rabbit skeletal muscle (100 µm in diameter) with a regular and repeated striation pattern. This pattern, due to the overlapping thick and thin filaments of the sarcomere is repeated along the length of the muscle fibre with a repeat length of 2.2 µm for a muscle at natural rest length, and provided a useful calibration system for any distortion of the windows. It also illustrated the absence of any significant compression of the muscle. The sarcomere was imaged under a pressure of 1 and 130 bar, the merged image is also shown. The two images are superimposable, indicating no change in the muscle structure and no distortion of the image due to optical artefacts. In fact, studies of muscle fibres, in which small-angle X-ray diffraction was used (Knight et al., 1993) show no change in the spacing of the filaments within the muscle fibre beyond that expected from the compression of water [isothermal compressibility =4.57×10^10^ m^2^ N^−1^ at 25°C, or ∼0.46% per 100 bar (Weast et al., 1984)].

Imaging porcine red blood cells (Fig. 3C) produced a similar result. The cells appear identical under pressure of 1 and 100 bar. A line profile through the same cell at the two pressures also appears identical, indicating no compression or deformity of the ∼4.5-µm cell and no discernible image distortion at the resolution limit. Consistent with this, when cells from a log-phase culture of fission yeast were mounted within the chamber, the application of 100 bar pressure had no instantaneous effect upon the size, shape or integrity of the living yeast cell when compared with cells imaged at normal atmospheric pressure (Fig. 3D). As cells are maintained in medium within the chamber, we tested the ability to follow nuclear division and growth of *S. pombe* cells expressing GFP-labelled histone Hht1 (hht-GFP:kanMX6, hereafter referred to as *hht-gfp*) (Fig. 3E). By using the chamber it was possible to follow growth and nuclear organisation through multiple rounds of the cell cycle, with generation times almost equivalent to those published for equivalent cells in liquid culture (Fantes and Nurse, 1977).

We next used the same strain to examine the precise impact pressure has upon the growth and cell cycle. Cells were mounted within the chamber on the imaging system, before increasing hydrostatic pressure of the medium to 100 bar. This pressure was maintained for 24 h, while cell growth and nuclear organisation were monitored (Fig. 3F). Although the overall cell-cycle time was equivalent during atmospheric and 100 bar pressure, a delay in commitment to mitosis was observed in cells kept at 100 bar compared to cells at normal pressure. Consistent with the above data, we observed an accumulation of long cells, which contained either a single nucleus (Fig. 3F) indicating a delay in mitotic progression, or two nuclei and a non-cleaved septum (Fig. 3F arrowhead). To ensure cells remained viable for the duration of this and subsequent experiments, cell growth was monitored upon return to a pressure of 1 bar at the end of the incubation (after 24 h). Consistent with a delay in M-phase progression, these longer cells went through a rapid round of cell division upon returning to atmospheric pressure (Fig. 3F, 1h and 2h post release). At 100 bar pressure there is no direct perturbation of protein structure, and this effect is most likely due to biochemical responses (e.g. changes in equilibria) within the cell.

**Fig. 4.**
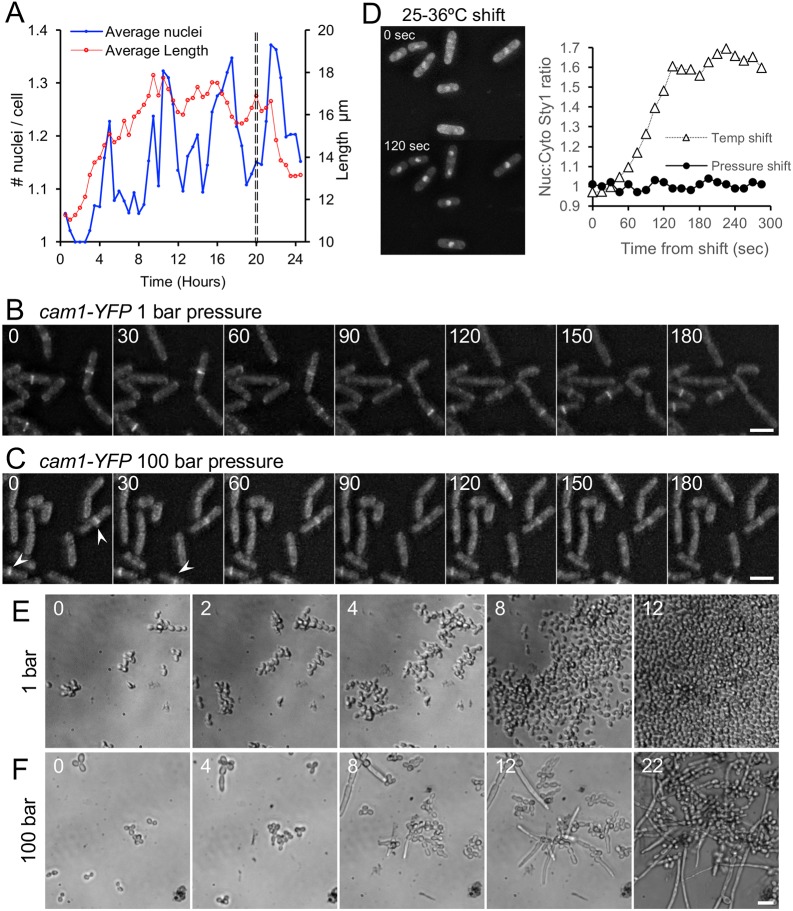
**Pressure of 100 bar reversibly alters cell-cycle progression in *S. pombe* and *C. albicans*.** (A) Graph showing the average change in cell length (red) and the average change in the number of nuclei per cell (blue) in *S*. *pombe* *hht-gfp* cells when cultured at 100 bar for 20 h, indicating that pressure induces synchronisation of cell cycle progression. The dashed vertical line indicates the time at which the pressure was reduced to 1 bar. For this representative experiment >300 cells were measured and analysed at each time point indicated. (B,C) Time-lapse images of *cam1-YFP* fission yeast cells mounted in the pressure chamber at 1 bar (B) or 100 bar (C). Images show pressure-induced accumulation of long cells with Cam1 foci accumulation (indicating polarised cell growth) at the cell equator (arrowheads). Numbers within images indicate the time (in min) exposed to pressure. (D) Nuclear import of the GFP-labelled MAP kinase Sty1 in response to temperature and pressure. While the ratio of nuclear:cytoplasmic Sty1-GFP signal (Nuc:Cyto) rapidly increased upon increasing temperature from 25 to 36°C (images and triangles in graph), increasing hydrostatic pressure from 1 to 100 bar had no discernable effect upon Sty1 distribution (filled circles) over the same time scale. (E,F) Time-lapse images showing the growth pattern of *C. albicans* cells cultured for up to 22 h in the pressure chamber at 1 bar – resulting in normal growth (E) – or 100 bar – resulting in decreased and switch to pseudohyphal growth (F). Scale bars: 10 µm.

To characterise the nature of the pressure that induced delay in cell division, the experiment was repeated, images were captured at multiple locations on the window every 30 min, and the average cell length and average number of nuclei per cell were calculated by measuring >300 cells at each time point (Fig. 4A). The mean cell length was consistently seen to increase for 10 hr when cells were subjected to 100 bar, but rapidly returned to normal length on pressure release (Fig. 4A, red line). Surprisingly, monitoring of the ratio between mono- and bi-nucleated cells revealed pressure-induced multiple rounds of synchronised nuclear division throughout the pressure chamber (Fig. 4A, blue line). To further examine this delay in cell-cycle progression, we used a strain expressing the Cam1, homologue of calmodulin fused to YFP (*cam1-YFP* cells) to allow simultaneous monitoring of spindle pole dynamics and actin-associated growth machinery. In contrast to *cam1-YFP* cells cultured within the chamber at 1 bar, which displayed a normal dynamic distribution of Cam1 (Fig. 4B, Movie 1), *cam1-YFP* cells at 100 bar pressure showed cytokinetic actomyosin rings that failed to constrict at the same rate as cells cultured at 1 bar pressure (Fig. 4C, arrowheads). In addition spindle poles failed to elongate and mitotic cells failed to progress beyond anaphase (Movie 2).

It has previously been reported that short 10 min bursts of significantly higher pressures (∼1000 bar) induce a MAP kinase stress response that can impact survival (George et al., 2007). To explore whether the non-toxic 100-bar-induced delays observed here were brought about by a similar activation of the stress response, we monitored nuclear shuttling of the MAP kinase Sty1. In the absence of stress Sty1 is normally cytoplasmic; however, within minutes of detecting stress, it accumulates within the nucleus to phosphorylate transcription factors in order to trigger a stress response (Gaits et al., 1998). Using an *S*. *pombe sty1-gfp* strain that expresses a Sty1-GFP fusion protein (Zuin et al., 2005), we followed Sty1 dynamics following either rapid increase in temperature or pressure within the chamber. While rapid increase in temperature from 25–36°C induced redistribution of Sty1 into the nucleus of cells, increasing pressure to 100 bar had no impact upon Sty1 localisation over the same time period, as it remained cytoplasmic (Fig. 4D).

To explore these findings further, we examined the pressure-induced retardation of *S. pombe* cell-cycle progression in strains that carried deletions in genes encoding the checkpoint pathway protein Sty1 (MAP kinase; *sty1*Δ), Wis1 (MAP kinase kinase; *wis1*Δ), Mad2 (spindle assembly checkpoint protein; *mad2*Δ) or Wee1 (negative regulator of mitosis; *wee1*Δ) (He et al., 1997; Russell and Nurse, 1987; Shiozaki and Russell, 1995; Warbrick and Fantes, 1991). Intriguingly, deletion of any one of these checkpoint and regulatory proteins had no significant effect (Student's *t*-test <50% level of significance) on the pressure-induced delay in cell-cycle progression, as measured by the relative increase in cell length after culturing cells at 100 bar for 20 h [ratio of pressure-induced difference in average cell length (*n*>200 cells per sample); wild type: 1.15; *sty1*Δ: 1.14; *wis1*Δ: 1.18; *mad2*Δ: 1.21; *wee1*Δ: 1.19] (data not shown). Together these data indicate the observed delays in cell-cycle progression are brought about by disruption in the integrity of normal cytoskeletal dynamics rather than inhibition in cell-cycle control.

In a final investigation into the effect of hydrostatic pressure on yeast growth dynamics, we investigated the impact pressure has upon the growth of a different yeast cell, the pathogenic budding yeast *Candida albicans*. Under standard growth conditions, *C. albicans* laboratory strains displayed a normal, vegetative budding-yeast-like, rapid growth pattern (Fig. 4E). However, when these cells were cultured at a pressure of 100 bar, we observed not only dramatic delay in growth but also a switch to pseudohyphal growth (i.e. cells became elongated, showed unipolar budding pattern, stayed physically attached to each other, invaded the growth substrate) (Fig. 4F), which was reversed on release of pressure (not shown).

## DISCUSSION

Here, we have described a simple to use moderately high-pressure fluorescence-imaging system that allows non-invasive and non-toxic monitoring of protein and organelle dynamics in living yeast cells. The system has the potential to find wide use at the interface between molecular and cell biology in living organisms as diverse as bacteria and mammalian cells, as well as in observing development in small metazoan organisms.

Applying changes in hydrostatic pressure has been widely used to study protein–protein, protein–ligand and protein–membrane interactions by using either purified proteins or the same proteins in intact cells (Demazeau and Rivalain, 2011). The ability to study the same molecular process using the same perturbation method with both isolated proteins and in cells provides an attractive and invaluable method to define the role of specific molecular events within cell physiology. However, while the ability to use fluorescent proteins and dyes to label molecules has enabled their location, colocalisation and redistribution to be examined in a living cell, the lack of a high-pressure live-cell imaging system has limited the use of pressure as a perturbation tool. The effects of pressure on cellular architecture have been studied by using fixed cells as, until recently, fluorescent imaging systems have not been used on live cells at high pressure. Here, we have described the analysis of individual proteins and organelles of cells at high pressure. We have shown that moderate changes in pressure have a benign effect on cells, report minimal effects a pressure of 100 bar has upon cell viability and on activation of their stress pathways. However, the same pressure perturbs the cell in several striking ways, slowing growth, inhibiting cell division and altering cell morphology. Dissecting which signalling pathways, cellular components and molecules are involved will now be possible.

There are clear advantages of using pressure to modulate the cell. The speed of application and release of the pressure (potentially within <1 ms) allows a sequence of events to be followed in real time. Crucially the easy reversibility of the effects of pressure allows us to define whether the same pathways operate during both inhibition and recovery of the pressure effect. A stable cell population can be repeatedly exposed to pressure changes without impacting cell viability.

By their nature, perturbation methods tend to make small changes to the system, such that only delicately poised equilibria or steady-states are affected. For example, it is well known that protein unfolding can be induced by exposure to high pressure; however, the protein needs to be poised near the transition between folded and unfolded state (by high temperature or the addition of organic solvent), i.e. before the modest pressures used here will induce any unfolding of most proteins. Similarly, the equilibrium between ‘on’ and ‘off’ states of a signalling system (calmodulin and/or troponin C, channel opening, G-proteins; see Conti et al., 1984; Eccleston et al., 1988; Pearson et al., 2008; Petrov et al., 2011) will only be perturbed when the system is poised between ‘on’ and ‘off’ states. For example, exposure to high pressure will activate muscle contraction when free Ca^2+^ is near the reaction equilibrium of the troponin C-binding reaction but not at high or low Ca^2+^ levels (Fortune et al., 1994). Thus, perturbation of a cell will depend upon which signalling pathways are operative at time of perturbation; i.e. the effects of pressure may be expected to be different in interphase versus cell division, during log-growth versus stress conditions or in stimulated versus non-stimulated cells.

There are many potential applications for this technology to not only further our understanding of mechanisms and molecular equilibria within a living cell, but also in the development of novel drug therapies. Moderate pressure allows the inducible disruption of the cytoskeleton and to have discrete effects on structures of different dynamic stability (e.g. at the cell surface versus within cytosol, stress fibres versus cortical actin, microtubule filaments versus spindle fibres).

It has long been established that the application of pressure can stall cell division in a wide variety of cells (Marsland, 1938; Salmon, 1975a,b; Salmon et al., 1976). Here, we have shown that this process is not only fully reversible but that it does not activate the stress response pathway. In addition, we also reported a reversible pressure-induced synchronisation of cell growth and division and that, interestingly, upon release to normal pressure, the whole cell population underwent a rapid round of cell division. This allowed us to examine bulk signalling within an entire population of cells.

Finally, the observation that modest increases in pressure (100–200 bar) can induce pseudohyphal growth in the pathogenic yeast *C. albicans* is consistent with previous studies describing that this growth state can be induced by disruption of actin cytoskeleton dynamics (Sudbery, 2011), and provides an attractive mechanism to screen for hyphal inhibitors to identify drug therapies that might prevent transition to the pathogenic invasive growth state.

## MATERIALS AND METHODS

### Cell culture

The fission yeast used in the study were prototrophic *cam1-YFP:kanMX6*, *hht-*GFP:kanMX6 (hht-GFP); sty1-GFP:kanMX6 (sty1-gfp); *sty1::URA4, mad2::URA4, wee1::URA4 wis1::URA4* and wild-type strains. *cam1-YFP* cells were generated as described previously (Bähler et al., 1998) using appropriate primers and template. All strains were backcrossed and validated prior to use. Cell culture and maintenance were carried out according to Moreno et al., 1991, using filter-sterilised Edinburgh minimal medium (EMM) containing glutamic acid as a nitrogen source (EMMG). The *Candida albicans* strain used is a derivative of the strain BWP17 (Wilson et al., 1999) *ura3*::imm434/*ura3*::imm434 *iro1*/*iro1*::imm434 *his1*::hisG/*his1*::hisG *arg4*/*arg4*, which was cultured in synthetic complete (SC) medium (Formedium, Hunstanton, UK). All cells were maintained in early to mid-log phase for 48 h before analysis. Early-log phase pre-conditioned minimal medium was used in all time-lapse experiments.

### Preparation of cell samples

Small-bundle muscle fibres were dissected from rabbit psoas muscle and membranes removed by treatment with detergent for 2 h (0.5% Brij-58; Sigma Aldrich) under relaxing conditions (70 mM propionic acid, 8 mM MgCl_2_, 5 mM EGTA, 7 mM ATP-Na_2_, 6 mM imidazole pH 6.8), and were then stored in 50% glycerol at −20°C until required as described by Knight et al. (1993). Porcine red blood cells were isolated from freshly drawn blood (sourced from a local abattoir) by centrifugation and washed three times with Tris-buffered isotonic saline (0.12 M KCl, 10 mM Tris, pH 7.4).

### Microscopy

Imaging was undertaken on an Olympus IX73 microscope with either LUCPLFLN 40×0.6NA, LUCPLFLN 60×0.7NA long-working-distance air lenses, LUMPLFLN 60× W 1.0NA water-immersion lenses or PLANAPO 60×1.4 NA oil-immersion lenses. Samples were illuminated using LED light sources (Cairn Research Ltd, Faversham, UK) with appropriate long-pass filters (Chroma, Bellows Falls, VT). Images were captured by using an Evolve EMCCD camera (Photometrics, Tucson, AZ), and the imaging system was controlled using Metamorph software (Molecular Devices, Sunnyvale, CA). Each 3D-maximum projection of volume data were calculated from *z*-plane images, spaced 0.5 μm apart, using Metamorph software.

### Standard pressure chamber

The effects of pressure on cell viability, length and generation time used a pressure chamber originally designed for collection of small-angle X-ray scattering data of muscle fibres (Knight et al., 1993). Hydrostatic pressure was applied to this chamber by using a Kontron Instruments 422 HPLC pump (Watford, UK) and controlled using Labview software (National Instruments, Austin, TX). While this chamber was maintaining stable pressures of >500 bar for several hours, its windows were unsuitable for optical imaging.

### High-pressure imaging chamber design

The cell design is shown in Fig. 2 and based on the design of a pressure chamber used for studying the effects of pressure on contracting muscle fibres (Fortune et al., 1991; Knight et al., 1993). Components of the imaging cell were built at Cairn Research Ltd (Faversham, Kent, UK) and in the University of Kent Engineering Workshop. It was milled from a single 6×6×3 cm block of 316-stainless steel (sourced from Orion Alloys Ltd, Harlow, Essex, UK) with a 3.5-cm diameter cylinder through the middle. The window mounts were inserted from opposite sides of this hole and each held in position by six stainless steel screws (M4). The upper window mount held a 10-mm-thick perspex window, which provided a pathway for transmitted light. The lower mount was designed specifically to match the shape of the objective lenses used for fluorescence observation and allowed the lens to approach a stainless-steel disc used to support the observation window. O-rings on the surface of the window mounts provided the pressure seal with the wall of the cylinder block. Ports allowed connection via standard high-pressure liquid chromatography (HPLC) tubing to the pressure line, and a manual HPLC valve (SSI 02-0120) allowed chamber flushing and pressure release. The chamber was flushed and hydrostatic pressure applied and maintained as above. Pressure was applied and maintained using a Kontron 422 HPLC pump.

The design of the window was a balance between the working distance, the pressure range used and the size of the window. In order to allow rapid assembly and disassembly of the chamber, and to optimise assembly for specific conditions, the window consisted of three parts. The window mount (described above), a 1-cm diameter glass disc forming the window and a 2-cm diameter supporting stainless steel disc used to set the diameter of the observation window. The window mount and the stainless steel disc had highly polished surfaces to facilitate a seal between each pair of surfaces, the disc and glass window were held in place by glue. To test window performance an acetone/cellulose glue (a mixture of acetone-disolved cellulose that had been allowed to evaporate to required viscosity) was used that allowed rapid replacement of window and disc. For longer term use the window components were fixed in place using Araldite epoxy-adhesive (Huntsman Advanced Materials, Switzerland). The shortest working distance at a pressure of 100 bar was achieved by using a 1-mm-thick stainless steel disc with 1-mm-diameter window apertures and a standard 8-mm-diameter circular quartz coverslip that was 0.5 mm thick. Higher pressures and larger diameters of observation window were possible by using thicker glass and/or stainless steel discs but only together with increased working distance and, hence, poorer optical resolution. Use of specialist materials for the windows (diamond or sapphire) may allow higher pressures and lower working distances but at a much higher cost.

### Mounting cells for observation within the chamber

Before use, the chamber was sterilised with alcohol, assembled and flushed through with sterile water and sterile pre-conditioned medium. Cells were then mounted (without centrifugation) directly onto lectin-coated (Sigma L2380; 1 mg/ml) prepared quartz discs. The chamber was reassembled with the quartz disc and mounted cells in place (Fig. 2), and pre-conditioned medium was pumped through the system until all air bubbles had been excluded from the chamber. The chamber was then fitted onto the imaging system described above.

## Supplementary Material

Supplementary information
