# Supplementary material for: A novel live-cell imaging system reveals a reversible hydrostatic pressure impact on cell-cycle progression
Source: J Cell Sci. 2018 Aug 6;131(15):jcs212167. doi: 10.1242/jcs.212167 (PMC6104828; doi:10.1242/jcs.212167)
Supplement: Supplementary information [file joces-131-212167-s1.pdf]

A

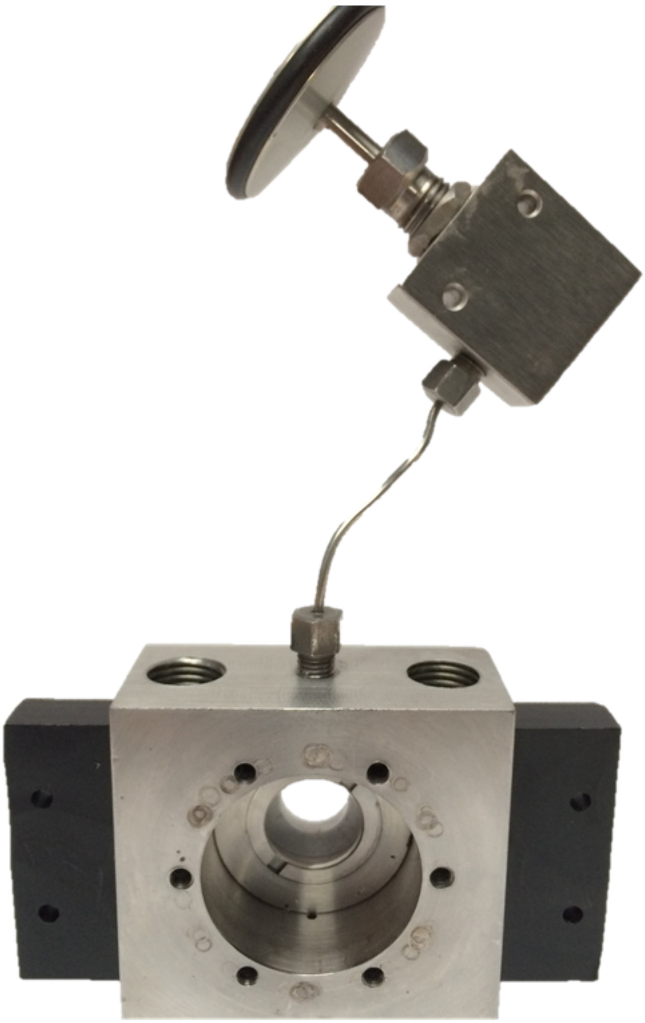

B

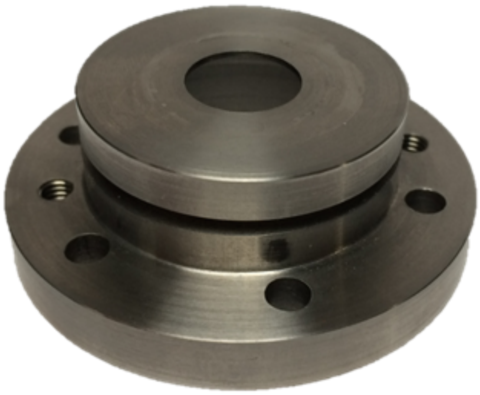

C

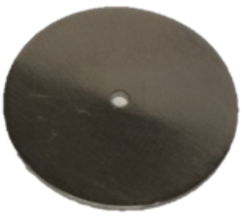

Journal of Cell Science • Supplementary information

Figure S1. Components of the high pressure live cell fluorescence microscopy chamber. (A) Main chamber with upper transmission light window fitted and release valve attached. (B) Lower observation window and (C) polished steel disc onto which is mounted the quartz coverslip.

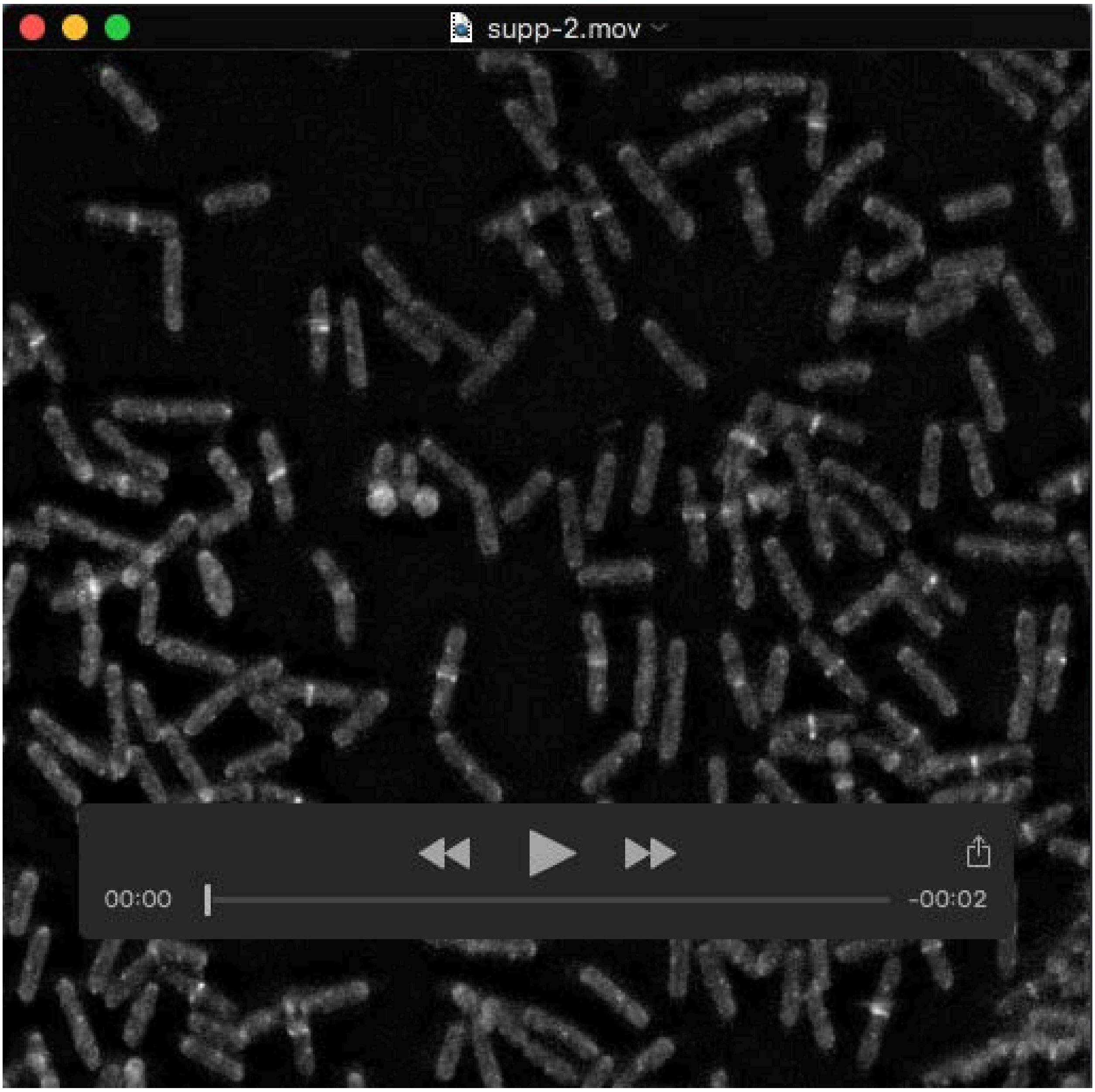

Movie S1. Cam1-YFP dynamics in *S.pombe* cells cultured in EMMG at 25ºC and 1 atmosphere pressure within the pressure chamber. YFP signal from mid-log phase *cam1.yfp* cells cultured within the pressure imagining chamber. Stage drift was not corrected. Images are maximum projections of 15 x 0.5µm spaced z-slice images, captured every 5 min.

Journal of Cell Science • Supplementary information

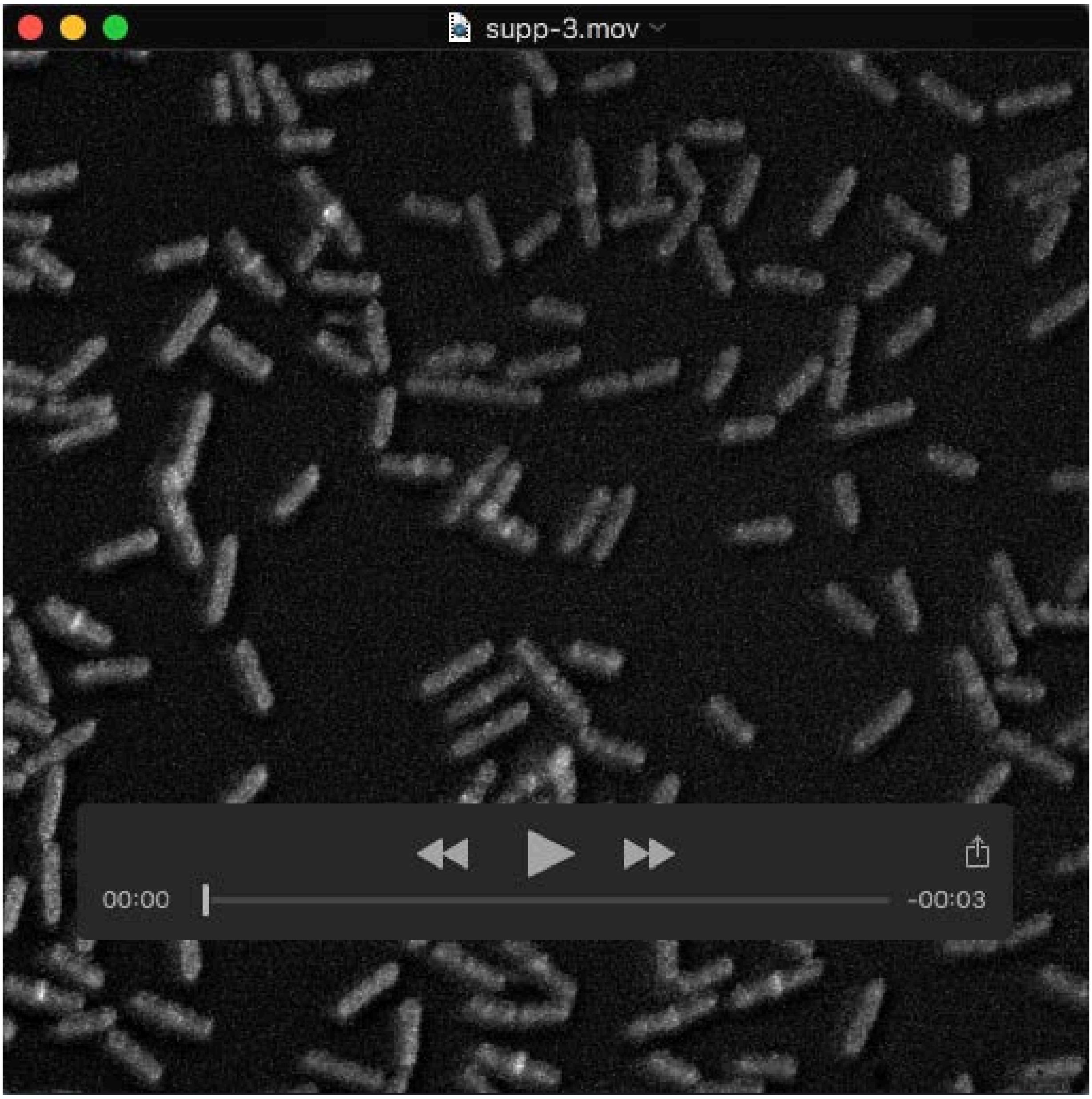

Movie S2. Cam1-YFP dynamics in *S.pombe* cells cultured in EMMG at 25ºC and 100 bar pressure within the pressure chamber. YFP signal from mid-log phase *cam1.yfp* cells cultured within the pressure imagining chamber. Stage drift was not corrected. Images are maximum projections of 15 x 0.5μm spaced z-slice images, captured every 5 min.
